# Executive Function and Spatial Cognition Mediate Psychosocial Dysfunction in Major Depressive Disorder

**DOI:** 10.3389/fpsyt.2018.00539

**Published:** 2018-10-29

**Authors:** Matthew J. Knight, Bernhard T. Baune

**Affiliations:** ^1^Discipline of Psychiatry, Adelaide Medical School, University of Adelaide, Adelaide, SA, Australia; ^2^Department of Psychiatry, Melbourne Medical School, University of Melbourne, Melbourne, VIC, Australia

**Keywords:** depression, MDD, cognition, spatial cognition, mediation, executive function, psychosocial functioning

## Abstract

**Background:** Cognitive and psychosocial dysfunction are prevalent and disabling features of Major Depressive Disorder (MDD). Emerging evidence suggests that poor cognitive functioning mediates the negative effect of MDD on psychosocial function. However, there is a lack of research examining the domain-specific nature of this relationship. The current study evaluated whether the relationship between MDD and specific psychosocial subdomains (e.g., autonomy, occupational functioning) was mediated by domain-specific cognitive deficits.

**Methods:** Data from 155 participants was obtained from the Cognitive Function and Mood Study (CoFaMS), a cross-sectional analysis of mood, cognition, social cognition, and functioning in individuals with MDD. Cognitive functioning was assessed (Current MDD *n* = 45, Healthy *n* = 110), with the Repeatable Battery for the Assessment of Neuropsychological Status (RBANS), the Colorado Assessment Tests (CATs), and the Psychology Experiment Building Language (PEBL). Psychosocial functioning was clinically evaluated with the Functioning Assessment Short Test (FAST).

**Results:** The results indicated that spatial cognition and executive functioning partially mediated the negative effect of MDD on overall psychosocial functioning, autonomy, and subjective cognition. In contrast, spatial and executive domains showed divergent mediation patterns on interpersonal relationships and leisure time.

**Conclusions:** The findings suggest that executive and spatial cognition play an important role in the pathology of overall psychosocial functioning, and specific functional issues in MDD. Treatments targeting psychosocial recovery in MDD may be improved by emphasizing executive and spatial cognitive remediation.

## Introduction

While major depressive disorder is characterized by impaired mood, psychosocial deficits are increasingly recognized as a core symptom of the illness (1, 2). Functional deficits have been identified in domains of occupational functioning, daily responsibilities, interpersonal relationships, financial management and self-perceived quality of life (1, 3–6). While individually debilitating, functional deficits also contribute substantially to the social and economic burden of disease (7, 8), with billions of dollars lost annually to functional issues related to MDD (9, 10). In addition, psychosocial deficits are frequently maintained despite improvement and remittance of mood symptoms (4, 11), and are associated with illness relapse and poor long-term recovery (1). The chronicity and clinical impact of psychosocial impairment highlights the need for an improved understanding of psychosocial dysfunction in MDD.

Emerging research suggests that cognitive deficits contribute independently to the development and maintenance of psychosocial dysfunction acutely depressed patients (6, 12–16). Cognitive dysfunction has been identified in domains of executive functioning, attention, working memory, learning, processing speed, and spatial cognition, with deficits in several domains linked to functional outcome (4, 16, 17). Cognitive impairment is typically greater in older MDD patients (18, 19), and in those with more severe depression (20), with recent evidence suggesting that cognitive deficits enhance functional issues in MDD (21). For example, research by Xiang et al. (21) identified that individuals with cognitive dysfunction and MDD reported poorer psychosocial functioning than individuals with cognitive dysfunction or MDD in isolation. Like psychosocial issues, cognitive deficits are frequently retained during the remitted stage of illness (22–24), and are associated with illness relapse (1, 25). The overlapping profile of psychosocial and cognitive dysfunction in MDD provides further support for the link between these dimensions of illness, and highlights the need for further investigation into cognitive dysfunction as a mechanism for functional difficulties in MDD.

A small body of research has identified that cognitive factors mediate the relationship between MDD and psychosocial functioning, suggesting that cognition plays an important role in the pathology of functional deficits (3, 11, 26–28). For example, Buist-Bouwman et al. (3) found that attention partially mediated the negative effect of MDD on role functioning (i.e., self-perceived ability to fulfill one's occupational and daily role) as reported by the International Classification of Functioning, Disability, and Health (ICF) (29). In addition, Kiosses and Alexopoulos (28) found that the relationship between MDD and impairments in daily living [i.e., Instrumental Activities of Daily Living (IADL)] was mediated by deficits in executive functioning. Taken together, these findings suggests that MDD leads to deficits across a number of cognitive domains (e.g., attention, executive functioning), and that these deficits in turn negatively impact psychosocial functioning. While the studied by Buist-Bouwman et al. (3) and Kiosses and Alexopoulos indicate a general mediation effect on overall (i.e., global) psychosocial functioning, there is a lack of research on which specific domains of psychosocial functioning are mediated by cognitive deficits in currently depressed patients. The IADL and ICF scales utilized in the above studies combine daily responsibilities (e.g., food preparation) and occupational functioning in a single composite indication of function, and as such do not differentiate between specific functional issues. Accordingly, it is not possible to conclude which domains of psychosocial functioning are negatively affected by cognitive dysfunction in MDD. Identification of domain-specific relationships in crucial, as adjunctive cognitive treatment should target cognitive domains which impact directly on patient functioning (2, 30).

**Figure 1:**
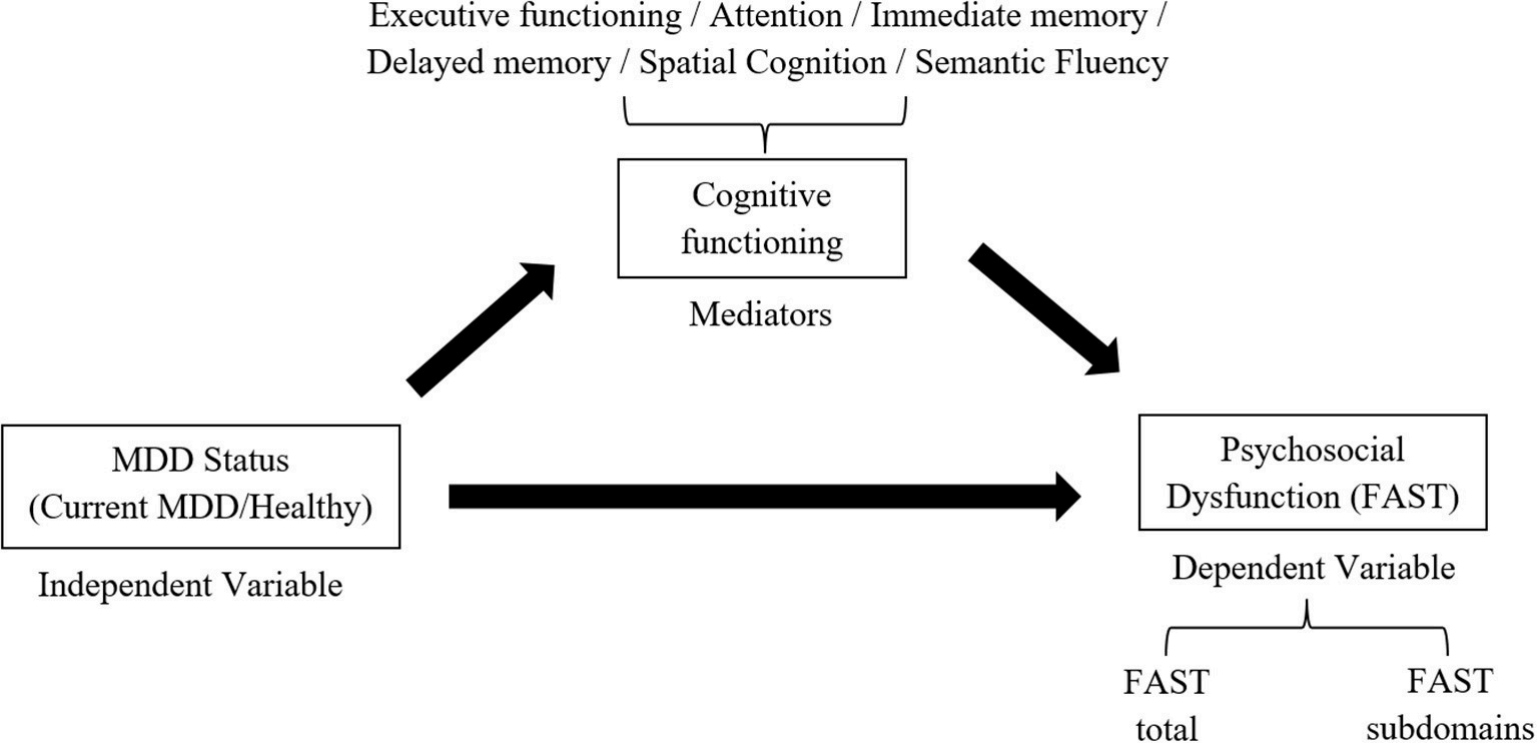
Proposed mediation relationship between MDD status (Current MDD, HCs), cognitive functioning, and psychosocial dysfunction.

The present research addresses the above gap in our understanding by evaluating the role of cognitive domains (e.g., executive functioning, attention, immediate memory) as mediators of specific psychosocial issues (e.g., autonomy, financial issues, interpersonal relationships). It was expected that cognitive deficits would mediate the negative effect of current MDD on psychosocial functioning. However, the limited research in this area did not allow domain-specific hypotheses regarding the effect of cognitive domains on specific psychosocial issues. Cognitive and psychosocial functioning was assessed in healthy, as well as acutely depressed patients, enabling detection of the MDD-Cognition-Psychosocial functioning relationship, illustrated in Figure 1. The primary aims of the present study were:

1. To evaluate whether the negative effect of a current episode of MDD on overall psychosocial functioning was mediated by performance in specific cognitive domains (i.e., executive functioning, attention, immediate memory, delayed memory, spatial cognition, semantic fluency).
2. To determine whether specific cognitive domains mediate the negative effect of a current episode of MDD on particular psychosocial issues (i.e., autonomy, occupational functioning, interpersonal relationships, financial issues, subjective cognitive dysfunction, leisure time).

## Method

Participant data (*N* = 155) for the current study was obtained from the Cognitive Function and Mood study (CoFaMS) (17); a cross-sectional analysis of cognition, mood, social cognition, and psychosocial functioning in persons with MDD. Participants were included on the basis of a current episode or previous diagnosis of depression following DSM-IV-TR criteria (31). Exclusion criteria were presence of a psychotic disorder, dementia, learning disorder, autism spectrum disorder, or other illnesses which can cause cognitive issues (e.g., brain tumor). Participants were selected from the CoFaMS study on the basis of completing standard assessments of cognitive and psychosocial functioning.

### Cognitive assessment

Cognitive assessments included the Repeatable Battery for the Assessment of Neuropsychological status (i.e., RBANS) (32), the Colorado Assessment Tests (i.e., CATs) (33), and cognitive tests in the Psychology Experiment Building Language (i.e., PEBL) (34). Psychosocial functioning was assessed with the Functioning Assessment Short Test (i.e., FAST) (35). Subjects were screened for mental illness with the MINI600 Neuropsychiatric Diagnostic Interview (36), as well a background medical and psychiatric history questionnaire. Participants with acute MDD were included (*n* = 45), as well as participants free of psychiatric illness (*n* = 110) [i.e., healthy controls (HCs)]. Individuals were defined as HCs if they had not experienced any of the 17 common illnesses included in the MINI600 (e.g., OCD, Anxiety disorders), and demonstrated HAM-D scores below 7 (37). Of those with current MDD, mean duration of illness prior to the study was 18 years. No age limit was included in CoFaMS, however all participants were at least 18 years of age. The mean age of participants was 36.29 (*SD* = 16.07), 61% were female, and mean years of education was 13.62 years (*SD* = 3.11). Student's *t*-tests and a chi-squared found identified no differences in age, sex, or years of education between the current MDD and HCs (*ps* > 0.05). Further demographic information stratified by MDD status (current MDD, HCs) is presented in Supplementary Table 1 in the Appendix.

Domain-specific cognitive functioning was obtained with RBANS, CATS, and PEBL test batteries. The RBANS is a neuropsychiatric screening tool designed as a brief assessment of cognitive performance in individuals with mental ilness (32). The current study employed the RBANS digit span, list learning, list recognition, store memory, figure recall, picture naming, figure copy, and line orientation tasks. Cognitive tests from the CATs battery included the *n*-back, visual span, word recall, tower of london, and Wisconsin card sorting tests (33). The PEBL is a freely available software package which includes many computerized cognitive assessments (e.g., Corsi blocks, mental rotation). The PEBL Sroop task and Wisconsin card sorting task^1^ were included in the current study. Domain-specific composite z-scores were derived from performance in the RBANS, CATS, and PEBL cognitive assessments, resulting in composite domains of executive functioning, attention, immediate memory, delayed memory, semantic fluency, and spatial cognition (15). The derivation of domain-specific composites by cognitive test scores is illustrated in Table 1 [see also Knight et al. (23)].

**Table 1:** Calculation of composite domain-specific *z*-scores by cognitive domain in the RBANS, CATS, and PEBL batteries.

**Cognitive domain**	**Cognitive tests**		
	**RBANS**	**CATS**	**PEBL**
Executive functioning		Tower of London (excess moves total) Wisconsin card sorting test (perseverative responses)	Stroop (incongruency errors) Tower of London (Total excess moves) Wisconsin card Sorting test (perseverative responses)
Attention	Digit span Coding	VSPAN^a^ (total average time) NBACK (accuracy)	Stroop (incongruency errors)
Immediate memory	List learning Store memory Digit span	Word Recall (total words recalled) VSPAN (correct trials forward) VSPAN (correct trials backward)	
Delayed memory	List recognition List recall Story recall Figure recall	Word recall (primacy total)	
Semantic fluency	Picture naming Semantic fluency	Word recall (total words recalled)	
Spatial cognition	Figure copy Line orientation	Tower of London (percentage above optimal)	

a *Vspan, Verbal memory span*.

### Assessment of functioning

Psychosocial functioning was assessed with the FAST, a clinician administered interview in which dysfunction is evaluated in six domains (i.e., autonomy, occupational functioning, cognitive functioning, financial issues, leisure time, and interpersonal relationships) (35). Impairment is measured by the interviewer on a four-point scale, with 0 indicating no difficulty and 3 severe impairment. Six composite FAST ratings were derived from the mean score of impairment in each functional subdomain [i.e., autonomy (*Range:* 0–12), occupational dysfunction (*Range:* 0–15), subjective cognitive dysfunction (*Range:* 0–15), financial issues (*Range:* 0–6), interpersonal relationships (*Range:* 0–18), and leisure time (*Range:* 0–6)]. Overall psychosocial functioning (i.e., FAST total score) was indicated by the sum of FAST subdomains (*Range*: 0–72). FAST total score and FAST subdomain composites were used as dependent variables in separate multiple regression analyses.

### Statistical analyses

Analyses were conducted with SPSS for windows, version 24. Regression analyses were performed to test whether the relationship between MDD status (Current MDD, HCs) and overall psychosocial functioning was mediated by domain-specific cognitive performance. Potential cognitive mediators were identified following the procedure established by Baron and Kenny (38), in which three regression equations are used to derive three criteria for mediation. In the first regression equation, the mediator (i.e., domain-specific cognition) was regressed on the IV (i.e., MDD status), in the second equation the DV (i.e., FAST total score) was regressed on the IV, while in the third equation the DV is regressed on the IV and mediator. To establish mediation three criteria must be met; (1) the IV must be significantly associated with the mediator in the first equation, (2) the IV significantly affects the DV in the second equation, and (3) the mediator must influence the DV in the third equation. If these relationships hold in the expected direction^2^ then the effect of the IV on the DV in model 3 must be reduced relative to model 2. Complete mediation occurs if the effect of the IV on the DV in equation 3 becomes non-significant with the inclusion of the mediator. Partial mediation occurs if the effect of the IV on the DV in equation 3 is reduced but remains statistically significant.

The procedure above for testing mediation was repeated six times, substituting as mediators each of the six composite cognitive domain z-scores (i.e., of executive functioning, attention, immediate memory, delayed memory, semantic fluency, and spatial cognition). This procedure enabled detection of whether specific cognitive domains mediated the effect of MDD status on overall psychosocial function (i.e., FAST total). If criteria (1) and (3) were satisfied for the included cognitive mediator, then this cognitive domain was further employed in subsequent analyses of FAST subdomains (i.e., FAST autonomy, occupational functioning, interpersonal relationships, leisure time, financial issues, subjective cognitive dysfunction). In contrast, those cognitive domains which did not satisfy criteria (1) and (3) did not proceed to psychosocial-subdomain specific mediation tests. Age, sex, and years of education were entered as covariates, as these factors can affect the relationship between cognitive and psychosocial functioning (4, 39).

## Results

### Mediation criteria–fast total score

Regression analysis revealed that mediation criteria (2) was satisfied for analysis of FAST total score, demonstrating a significant effect of MDD status (current MDD coded 1, HCs coded 0) on FAST total score (*p* < 0.001). The results indicated that spatial cognition satisfied criteria (1) and (3), with spatial cognition negatively associated with MDD status (*p* = 0.028) and FAST total score (*p* = 0.031). Mediation criteria (1) and (3) approached significance for executive functioning, such that executive cognition was associated with MDD status (*p* = 0.054) and FAST total score (*p* = 0.063). Mediation analyses for spatial cognition and executive functioning are reported separately below. For all remaining composite cognitive domains (i.e., attention, immediate memory, delayed memory, semantic fluency), either criteria (1) or criteria (3) did not approach significance. Inferential statistics for mediation criteria (1), (2), and (3) are reported in Table 2.

**Table 2:** *P-*values for mediation criteria, and *R*^2^ values, of the MDD status-FAST total relationship as mediated by composite cognitive domains (*N* = 155).

**Cognitive domains**	**Mediation criteria**			**Change in** ***R***^**2**^ **owing to mediator**	
	**Criteria (1)**	**Criteria (2)**	**Criteria (3)**	**Equation 2 R^2^**	**Equation 3 R^2^**
Executive functioning	0.054	<0.001	0.063	0.240	0.225
Attention	0.445	<0.001	0.002	NA	NA
Immediate memory	0.449	<0.001	0.004	NA	NA
Delayed memory	0.465	<0.001	0.004	NA	NA
Semantic fluency	0.065	<0.001	0.116	NA	NA
Spatial cognition	0.028	<0.001	0.031	0.240	0.229

*NA, Not applicable (no mediation); Criteria (1), IV associated with mediator; Criteria (2), IV associated with DV; Criteria (3), Mediator associated with DV. Linear regression model adjusted for age, gender, and years of education*.

### Spatial cognition

Before entering spatial cognition (i.e., equation 2), MDD status explained a significant 24% of variance in FAST total score (*R*^2^ = 0.24, *p* < 0.001). After controlling for the contribution of spatial cognition in the third equation, MDD status explained a lower, but still significant, 22.9% of variance in FAST total score (*R*^2^ = 0.229, *p* < 0.001), indicating that spatial cognition partially mediated the effect of MDD status on FAST total score. Subsequent tests using FAST subdomains as dependent variables, rather than FAST total score, indicated that spatial cognition satisfied criteria (1), (2), and (3) in subdomains of interpersonal relationships, subjective cognitive dysfunction, and autonomy^3^ (see Table 3). For interpersonal relationships, variance explained by MDD status was reduced from 28 to 26.4% by the inclusion of spatial cognition in equation three. The variance in subjective cognition explained by MDD status was reduced from 24.3 to 23.4% by the inclusion of spatial cognition, while the variance explained in autonomy by MDD status was reduced from 17.9 to 17.1%. In all analyses, the direct effect of MDD status on FAST subdomains remained significant (*p* < 0.001) after controlling for spatial cognition, indicating partial mediation.

**Table 3:** *P-*values for mediation criteria, and *R*^2^ values, for the MDD status-FAST Subdomain relationship as mediated by spatial cognition (*N* = 155).

**FAST subdomains**	**Mediation criteria**			**Change in** ***R***^**2**^ **owing to mediator**	
	**Criteria (1)**	**Criteria (2)**	**Criteria (3)**	**Equation 2 R^2^**	**Equation 3 R^2^**
Autonomy	0.028	<0.001	0.068	0.179	0.171
Occupational functioning	0.028	<0.001	0.200	NA	NA
Subjective cognition	0.028	<0.001	0.028	0.243	0.234
Leisure time	0.028	<0.001	0.103	NA	NA
Financial issues	0.028	0.319	0.103	NA	NA
Interpersonal relationships	0.028	<0.001	0.014	0.280	0.264

*NA, Not applicable (no mediation); Criteria (1), IV associated with mediator; Criteria (2), IV associated with DV; Criteria (3), Mediator associated with DV. Linear regression model adjusted for age, gender, and years of education*.

### Executive functioning

For analysis of FAST total score, including executive functioning in the third equation reduced the variance explained by MDD status (current MDD, HCs) from 24 to 22.5%. However, the direct effect of MDD status remained significant (*p* < 0.001) in equation 3, indicating partial mediation by executive functioning. Following regression analyses with FAST subdomains as DVs demonstrated that criteria (1), (2), and (3) were met for autonomy, subjective cognitive dysfunction, and leisure time (see Table 4). Analysis of autonomy indicated that MDD status explained 17.9% variance in equation 2, while the inclusion of spatial cognition as a mediator in equation 3 reduced variance explained by MDD status to 16.3%. MDD status explained 24.2% of the variance in subjective cognitive dysfunction in equation 2, whereas a lower 22.4% of variance was explained by MDD status after controlling for executive functioning in equation 3. Variance in leisure time explained by MDD status was reduced from 15% in equation 2 to 13.2% by the inclusion of executive functioning in equation 3. The direct effect of MDD status on FAST subdomains remained significant in equation 3 in all analyses (*p* < 0.001), indicating partial mediation of autonomy, subjective cognitive dysfunction, and leisure time by executive dysfunction.

**Table 4:** *P-*values for mediation criteria, and *R*^2^ values, for the MDD status-FAST Subdomain relationship as mediated by executive functioning (*N* = 155).

**FAST subdomains**	**Mediation criteria**			**Change in** ***R***^**2**^ **owing to mediator**	
	**Criteria (1)**	**Criteria (2)**	**Criteria (3)**	**Equation 2 *R*^2^**	**Equation 3 *R*^2^**
Autonomy	0.054	< 0.001	0.023	0.179	0.163
Occupational functioning	0.054	< 0.001	0.471	NA	NA
Subjective cognition	0.054	< 0.001	0.049	0.242	0.224
Leisure time	0.054	< 0.001	0.018	0.150	0.132
Financial issues	0.054	0.319	0.015	NA	NA
Interpersonal relationships	0.054	< 0.001	0.152	NA	NA

*NA, Not applicable (no mediation), Criteria (1), IV associated with mediator; Criteria (2), IV associated with DV; Criteria (3), Mediator associated with DV. Linear regression model adjusted for age, gender, and years of education*.

## Discussion

The current study examined whether the detrimental effect of a current episode of MDD on psychosocial functioning was mediated by deficits in cognitive functioning. Analyses of overall psychosocial functioning (i.e., FAST total) indicated that spatial cognition and executive functioning partially mediated the negative effect of current MDD on psychosocial functioning. In contrast, attention, immediate memory, delayed memory, and semantic fluency did not mediate psychosocial functioning. Follow-up analyses with spatial cognition and executive functioning as mediators of specific psychosocial issues (e.g., autonomy) indicated overlapping patterns of mediation in domains of autonomy and subjective cognition. In contrast, only spatial cognition mediated deficits in interpersonal relationships, while executive functioning alone mediated issues in leisure time. These findings provide new evidence that executive and spatial cognition play an important role in the pathology of specific psychosocial issues in current MDD.

To our knowledge, these results are the first to demonstrate that specific psychosocial issues, as opposed to overall functioning, are negatively affected by specific cognitive domains (3, 28). The overlapping mediation patterns in autonomy and subjective cognition suggest that these functional domains may be more sensitive to objective cognitive deficits than other functional issues (e.g., financial management), which may be primarily affected by other MDD symptoms (e.g., pessimism, social isolation). Given the primacy of autonomy and subjective cognition in the current results, it is worthwhile discussing the contribution of executive and spatial cognitive domains to these issues.

The role of executive functioning in autonomy is likely explained by the broad application of executive functions in a number of behavioral and cognitive abilities (e.g., forward planning, problem solving) (2, 22, 23), which have been shown to contribute to taking responsibility for daily duties, and maintaining functional independence (e.g., hygiene, grocery shopping) (19, 35). Losses in spatial cognition may negatively affect autonomy by increasing the probability of spatial errors and oversights (e.g., disorientation, driving errors), which could negatively affect self-confidence in maintaining autonomy, particularly in acutely depressed patients who are typically hypersensitive internalization of errors (40). Similarly, the negative effect of executive and spatial cognition on subjective cognitive dysfunction may be the result increased cognitive failures (e.g., missing appointments, forgetting names, failure to solve problems), which are associated with poor cognitive functioning (41) and greater self-perception of cognitive dysfunction (42).

It is noteworthy that deficits in spatial cognition, but not executive functioning, mediated deficits in interpersonal relationships. While deficits in spatial cognition have been noted in MDD (12), and in global functioning (43), their contribution to the pathology of deficits in interpersonal issues has not previously been identified. It is possible that spatial cognitive skills contribute to several indirect features of social interaction (e.g., arriving on time, personal space, physical contact). While these indirect spatial factors are a plausible mechanism for interpersonal issues in current MDD, spatial cognition may also be reflective of more complex cognitive processes related to social abilities. Specifically, some research has suggested that spatial skills are employed in visualization of social-cognitive concepts (e.g., in/out-groups, social constraint, “better/worse”) (44, 45), which could also explain the contribution of spatial skills in maintenance of interpersonal relationships. These findings point to the importance of spatial skills in acutely depressed patients, and call for further research on the role of spatial cognition in social/interpersonal functioning.

Executive functioning, but not spatial cognition, mediated the negative effect of current MDD on leisure time. Leisure time may be reliant on executive functioning due to the necessity to plan hobby/personal interest time effectively, balance work/leisure experiences, and maintain focus on leisure activities. Executive inhibition may be particularly crucial for maintenance of leisure time in MDD patients, as cognitive inhibition is key to blocking negative intrusions which can interfere with enjoyment of leisurely activities in currently depressed patients (46, 47). Specifically, failure to inhibit negative thoughts may result in negative material consuming cognitive resources in working memory and attention, decreasing engagement and focus in leisure activities (48). These findings suggest that cognitive treatments targeting executive functioning may be particularly beneficial for patients demonstrating loss of interest/motivation in leisure activities.

Additional findings of interest in the current study were the lack of mediation relationships detected for domains of immediate/delayed memory, attention, and semantic fluency. While several studies have demonstrated that poor cognitive function in these domains is associated with psychosocial dysfunction in depressed patients (4, 14), the present findings suggest that these associations do not play a major role in the mechanistic pathway between depressive illness and psychosocial dysfunction. This interpretation is supported by the current data, which demonstrated that immediate/delayed memory and attention were associated with psychosocial function, but not with depressive illness. Semantic fluency was not associated with depression or functional status, suggesting this domain may play a lesser role in functional and clinical status.

Broadly, the current results indicate that spatial cognition and executive functioning play an important role in functional disability in MDD. While cognitive dysfunction has been associated with functional deficits in general, these results are the first to demonstrate distinct mediation relationships between specific cognitive domains and specific psychosocial deficits. The domain-specific nature of our findings provide clear avenues for future research, which could investigate the roles of spatial cognition and executive function in psychosocial function in clinical subgroups (e.g., elderly, comorbid anxiety). Future research on this topic should also examine the pathological contribution of cognitive domains not included in the current study. For example, processing speed has been linked with major depression (49, 50), but could not be independently derived from cognitive tests in the CoFaMS study, and hence was not included in the present mediation analyses. Clinically, our results suggest that depressed patients with spatial cognitive or executive dysfunction should also be screened for functional deficits particularly in domains of leisure time and interpersonal relationships. Patients with cognitive dysfunction in these domains may also benefit broadly from cognitive training to remediate spatial cognitive or executive deficits (51).

It should be acknowledged that the present findings were drawn from a relatively modest sample size (*N* = 155), and hence require further validation with a larger and equally well characterized clinical population. In addition, the current findings should be replicated with more objective performance-based indications of psychosocial functioning [e.g., the Social Skills Performance Assessment (SSPA)] (52). While the FAST provided a comprehensive and domain-specific indication of psychosocial functioning, its results were derived from clinicians' judgment, and are hence vulnerable to clinician bias, highlighting the need for replication by performance-based measures (e.g., the SSPA). Further validation with self-reported measures of psychosocial function (e.g., the 36-Item Short Form Health Survey) would also be valuable. However, care should be taken when interpreting self-reported functional outcomes, which may be more closely associated with severity of mood symptoms than clinical interviews or objective measures (14).

Taken together, the current findings provide new evidence for the roles of executive and spatial cognitive dysfunction in psychosocial deficits in current MDD. Specifically, our findings suggest that MDD leads to deficits in executive and spatial cognition, which both contribute to functional issues in domains of autonomy and subjective cognitive dysfunction, and contribute differentially to leisure time, and social relationships. Emerging cognitive and adjunctive treatments for acute depression should consider executive and spatial domains as prime treatment targets (30), particularly in patients representing with issues in the psychosocial domains indicated above.

## Ethics statement

This study will be carried out in accordance with the recommendations of the NHMRC national statement on ethical conduct in human research, Royal Adelaide Hospital HREC. The protocol was approved by the Royal Adelaide Hospital HREC and the University of Adelaide HREC. All subjects gave written informed consent in accordance with the Declaration of Helsinki.

## Author contributions

BB coordinated and supervised the CoFaMS study, from which the current results were derived. MK and BB mutually developed the hypotheses and conceptual outline of the present manuscript. MK analyzed data and wrote the manuscript, while BB edited and provided feedback during the writeup.

### Conflict of interest statement

BB received speaker/consultation fees from: AstraZeneca, Lundbeck, Pfizer, Takeda, Servier, Bristol Myers Squibb, Otsuka, and Janssen-Cilag. The remaining author declares that the research was conducted in the absence of any commercial or financial relationships that could be construed as a potential conflict of interest.

## Footnotes

^1^The PEBL Card Sorting Test is a digital version of the original Wisconsin Card Sorting Test named Berg's Card Sorting Test, which is freely available and includes modified instructions suitable for the digital format.

^2^Predicted direction given variable coding: IV negatively related with mediator, IV positively related with DV, mediator negatively related with DV.

^3^Criteria 3 for was marginally significant for autonomy (see Table 3).
