# Supplementary material for: Executive Function and Spatial Cognition Mediate Psychosocial Dysfunction in Major Depressive Disorder
Source: Front Psychiatry. 2018 Oct 29;9:539. doi: 10.3389/fpsyt.2018.00539 (PMC6215806; doi:10.3389/fpsyt.2018.00539)
Supplement: Supplementary file 1 [file Table_1.DOCX]

| **Supplementary eTable 1.** | | | |
| --- | --- | --- | --- |
| Demographic characteristics by MDD status (Total, Current MDD, Healthy Controls). | | | |
|  | Total Sample (N= 155) | Current MDD (n= 45) | Healthy  (n= 110) |
|  |  |  |  |
| Gender |  |  |  |
| Female | *n*= 95 (61%) | *n*= 33 (73%) | *n*= 62 (56%) |
| Male | *n*= 60 (39%) | *n*= 12 (27%) | *n*= 48 (44%) |
| Age | *M*= 36.29 (*SD*= 16.07) | *M*= 38.58 (*SD*= 16.57) | *M*= 34.00 (*SD*= 16.57) |
| Years of Education | *M*= 13.62 (*SD*= 3.11) | *M*= 13.42 (*SD*= 2.66) | *M*= 13.71 (*SD*= 3.29) |
| HAM-D score | *M*= 13.29 (*SD*= 6.12) | *M*= 19.87 (*SD*= 5.21) | *M*= 6.7 (*SD*= 7.03) |
| Lifetime presence of Anxiety disorder | *n*= 29 (19%) | *n*= 29 (64%) | 0 (0%) |
| Lifetime presence of Bipolar disorder | *n*= 1 (0.6%) | *n*= 1 (2%) | 0 (0%) |
